# Oxidation kinetics of YBaCo_4_O_7+*δ*_ and substituted oxygen carriers

**DOI:** 10.1098/rsos.180150

**Published:** 2018-06-20

**Authors:** Limin Hou, Qingbo Yu, Kun Wang, Qin Qin, Mengqi Wei, Fan Yang

**Affiliations:** School of Metallurgy, Northeastern University, No. 11, Lane 3, Wenhua Road, He Ping District, Shenyang 110819, Liaoning, People's Republic of China

**Keywords:** oxidation, kinetics, low temperature, oxygen carriers

## Abstract

In this paper, the relaxation kinetics of the oxidation process of the YBaCo_4_O_7+*δ*_, Y_0.95_Ti_0.05_BaCo_4_O_7+*δ*_ and Y_0.5_Dy_0.5_BaCo_4_O_7+*δ*_ oxygen carriers is studied with isothermal reaction data. XRD analysis for fresh samples shows that all the samples have YBaCo_4_O_7+*δ*_ structure. Scanning electron microscopy images of samples show that the samples consist of porous agglomerates of primary particles. Isothermal TG experiments are conducted with temperatures of 290°C, 310°C, 330°C and 350°C, respectively. It is found that the Avrami-Eroféev model describes solid-phase changes in the oxygen absorption process adequately. The results show that the distributed activation energies of the oxidation process obtained by the Avrami-Eroféev model are 42.079 kJ mol^−1^, 42.944 kJ mol^−1^ and 41.711 kJ mol^−1^ for the YBaCo_4_O_7+*δ*_, Y_0.95_Ti_0.05_BaCo_4_O_7+*δ*_ and Y_0.5_Dy_0.5_BaCo_4_O_7+*δ*_ oxygen carriers, respectively. The kinetic model was obtained to predict the oxygen carrier conversion of oxygen absorption for different time durations. The kinetic parameters obtained here are quite vital when this material is used in reactors.

## 1. Introduction

It is generally accepted that carbon dioxide (CO_2_) emission is the main contributor to global warming. Oxygen-enriched combustion, one of the possible options to reduce CO_2_ emission, is not applied widely in industry due to the high cost of oxygen production. The process of chemical looping air separation (CLAS) was developed by Moghtaderi & Song in 2010 [1]. The process saves 74% of the power of the cryogenic air separation process [1]. The schematic of the CLAS process is described elsewhere [2]. The oxygen carrier circulates between the oxidation reactor and the reduction reactor. In the oxidation reactor, the oxygen carrier is fully oxidized by oxygen. In the reduction reactor, the oxygen carrier is fully reduced by steam or CO_2_.

Oxygen carrier materials are mainly those of metal oxide, perovskite and sulfate. The metal oxide oxygen carriers such as those that are Cu-based, Co-based and Mn-based have attracted the interest of researchers [3–13]. Perovskite oxygen carriers such as Ca_1−*x*_Pr*_x_*MnO_3−*δ*_, SrCoFe_3−*δ*_ and LaFe_1−*x*_Mn*_x_*O_3_ also have been investigated [14–16]. Zhao *et al*. [15] found that Mn substitution in LaFeO_3_ not only was conducive to the partial oxidation of CH_4_, but also enhanced the lattice oxygen mobility from the bulk to the surface of the oxygen carrier. Because of a low melting point and serious agglomeration of metal oxide, various support materials such as ZrO_2_, TiO_2_ and SiO_2_ have been explored [11,17,18]. Wang *et al*. [19] reported that the reduction rate of the combined CuO/Mn_2_O_3_ oxygen carrier with ZrO_2_ as a binder increased with increasing reduction temperature. Wang *et al*. [20] found that the Fe_2_O_3_ oxygen carrier had an effective impact on the conversion of typical bituminous coal in a chemical looping combustion system. Whitty & Clayton [21] reported that the activation energy of the oxidation of the CuO oxygen carrier with ZrO_2_ as a binder was 202 kJ mol^−1^. Arjmand *et al*. [12] found that the activation energy of reduction reaction of the CuO oxygen carrier was 313 kJ mol^−1^. Hossain found that the reduction kinetics of the NiO/Ce-γAl_2_O_3_ oxygen carriers was favourably expressed by the nucleation and crystal growth model. The estimated energy of activation for the reduction process was found to be in the range of 52–55 kJ mol^−1^ [22]. Zhu *et al*. found that the reduction characteristics of oxygen carriers of Fe_2_O_3_–60 wt%/Al_2_O_3_ had an impact on the efficiency of the chemical looping hydrogen generation process. Fe_3_O_4_–FeO was determined as the rate-limiting step with a lower reaction rate constant and a higher activation energy [23]. Hossain & de Lasa [24] found that the nucleation and nuclei growth model provided a better description of the reduction process for CoO–NiO/*α*Al_2_O_3_ oxygen carriers. Li *et al*. [25] found that a moving-bed reducer showed better performance than a fluidized-bed reducer for the syngas chemical looping process.

There has been extensive reporting in the literature on metallic oxides; unfortunately, industrial applications of metallic oxides consume a large amount of energy as their reaction temperatures are high. Hence investigations with oxygen carriers, which can react at low temperatures and are extremely time-efficient. It is excellent that the waste heat of low temperatures can be used as heat resources as the waste heat resources have not been used effectively. YBaCo_4_O_7+*δ*_ (donated Y114 phase) was synthesized originally by Valldor & Andersson in 2002 [26]. Karppinen *et al.* reported that YBaCo_4_O_7+*δ*_ experienced two processes: oxygen intake and release (the first being around 200–400°C, the other around 600–900°C) when heated to 1100°C in an oxygen-containing atmosphere. The first process was reversible with no decomposition of the YBaCo_4_O_7+*δ*_ phase [27]. The maximum oxygen content was obtained at temperatures lower than 500°C, achieving *δ* ≈ 1.0 and *δ* ≈ 1.2 in air and oxygen atmospheres, respectively [27,28]. The unique ability of the YBaCo_4_O_7+*δ*_ phase to reversibly absorb and release oxygen makes it a possible candidate as an oxygen carrier that works at low temperatures in the CLAS system. The crystal structure consists of the three-dimensional network of corner-sharing CoO_4_ tetrahedra. The corner-sharing CoO_4_ framework allows oxygen modification in an atomic arrangement. As YBaCo_4_O_7+*δ*_ decomposition takes place at a temperature above 600°C, improving that dynamical stability is a critical issue [29]. Doping in the Y or Co site is one of the positive choices to improve the stability. Ca, Tb-Lu and Zr can partially or completely substitute Y [28–35]. Fe, Al, Ga, Mn, Ni, Cu and Zn can partially substitute Co [36–38]. Kadota *et al.* [29] identified that the phase-decomposition temperature of RBaCo_4_O_7+*δ*_ increased with decrease in the radius of the R ion. The decomposition temperature increased with increase in Sr doping concentration [35]. The phase-decomposition temperature of the 114 phase was increased by Al, Ga and Zn substituting for Co [33,35–37]. The increase was prominent, especially for the samples substituted by Al and Ga [35]. Räsänen *et al.* [36] reported that Al and Ga co-substituting for Co was more favourable than a single substitution of Al or Ga for improving thermal stability. However, Fe and Al co-substitution weakened the effects of Al substitution [31].

Very few works in the current literature focus on the kinetics of YBaCo_4_O_7+*δ*_ oxygen carriers for CLAS applications. As is known, YBaCo_4_O_7+*δ*_ shows a slower oxygen absorption rate and a faster oxygen desorption rate at lower temperatures [32,39]. For the application, the kinetics features are greatly impacted by the reactor size and the solid inventory. The reaction rate of YBaCo_4_O_7+*δ*_ varies with different working parameters such as reaction temperature, conversion range, oxygen concentration and particle size [40–42]. Generally, the kinetics of gas–solid reactions is complex. However, for YBaCo_4_O_7+*δ*_ oxygen carriers, the kinetic description of the process is relatively simple as there is no phase change. Indeed, the product of the gas–solid reaction is of a different solid phase from that of the solid reactant, the difference in density of the two solid phases imposing chemical constraints on the solid–solid interface [43,44]. As this surface area is a kinetic parameter for the gas–solid reaction, a phenomenological kinetic description of the process is often impossible. In the case of perovskite, the oxidation process involves physical adsorption on the surface and the oxygen vacancies are filled by oxygen ions migrating from the bulk. The oxygen ions are involved in dissociative adsorption and chemical adsorption. The diffusion of oxygen ions is one of the processes which might control the rate of the oxidation reaction. The different adsorption steps and the possible surface migration of these adsorbed species to the reaction sites might also be rate-controlling [43]. In the case of the chemical reaction-controlled process, action there proceeds uniformly throughout the solid particles. In this case, different models need to be employed. The mechanism and kinetic parameters obtained here are quite vital when this material is used in the reactors.

## 2. Experimental section

### 2.1. Preparation of materials

Samples of YBaCo_4_O_7+*δ*_, Y_0.95_Ti_0.05_BaCo_4_O_7+*δ*_ and Y_0.5_Dy_0.5_BaCo_4_O_7+*δ*_ were synthesized by a solid-state reaction. Mixed appropriate stoichiometric amounts of the starting materials, Y_2_O_3_, TiO_2_, Dy_2_O_3_, BaCO_3_ and Co_3_O_4_, were ground thoroughly and then calcined at 1000°C for 15 h. The calcined samples were reground and calcined at 1100°C for 30 h. After calcination, all the samples were ground with a mortar and sieved with a 400-mesh sieve (average particle size less than or equal to 37.5 µm) for experiments and kinetic analysis.

### 2.2. Characterization of materials

Phase composition was studied by a powder X-ray diffraction technique (Panalytical, PW 3040/60; X'Pert Pro system with Cu K*α* radiation). X-ray data were recorded with a step scan of 0.02° for 2*θ* between 10° and 70°, and the cell parameters were determined with jade software. The microstructure of the synthesized samples was observed with scanning electron microscopy (SEM) on an ultra plus field emission scanning electron microscope. The oxygen absorption behaviour was observed with isothermal TG experiments in a thermogravimetric analyzer-TGA (STA409PC). During the TG experiment, a powder sample, with a mass of 10 mg, was heated to the target temperature (290, 310, 330 and 350°C) in a N_2_ atmosphere to prevent the occurrence of oxygen absorption. Then the atmosphere was changed to an air flow of 40 ml min^−1^, keeping the target temperature for 2 h to investigate the oxygen absorption behaviour. Before the kinetic experiments, the internal and external diffusion were eliminated by the experiments by varying the gas flow rate and the sample loading weight in the ranges of 20–40 ml min^−1^ and 10–20 mg, respectively.

## 3. Results and discussion

### 3.1. Characterization

**Figure 1.**
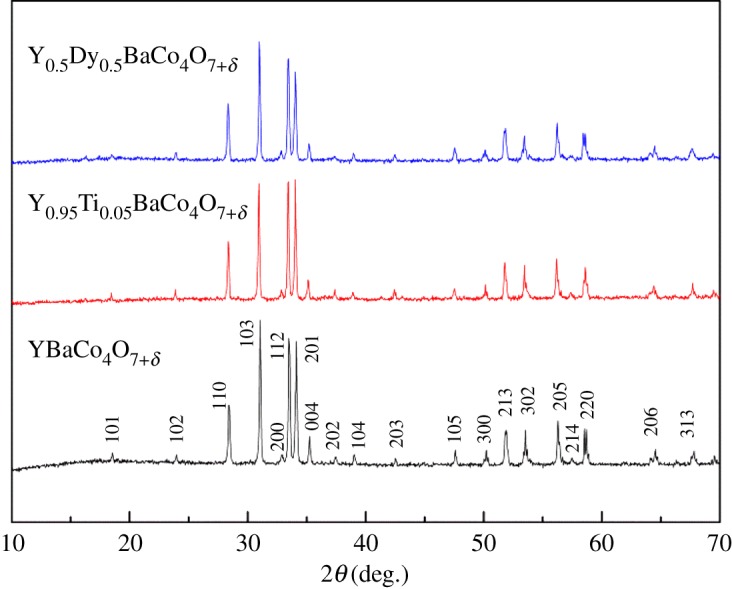
The XRD of the YBaCo_4_O_7+*δ*_, Y_0.95_Ti_0.05_BaCo_4_O_7+*δ*_ and Y_0.5_Dy_0.5_BaCo_4_O_7+*δ*_ samples.

**Figure 2.**
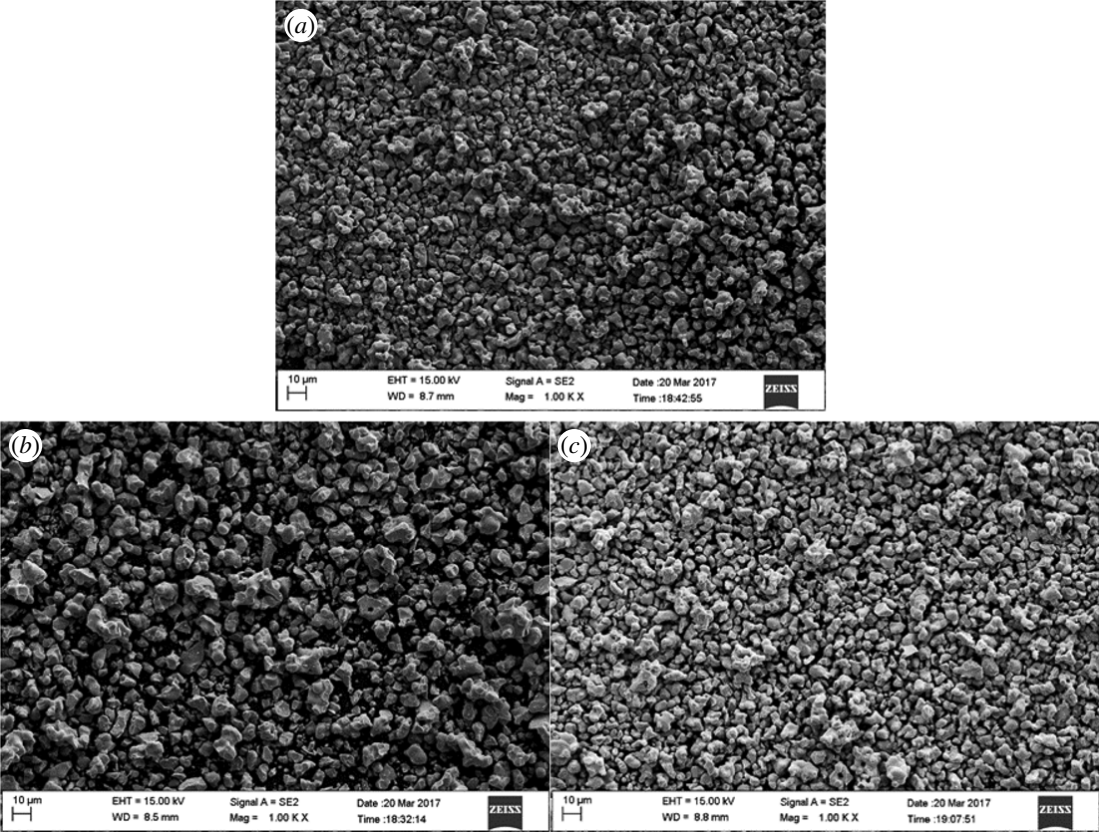
SEM images of the (*a*) YBaCo_4_O_7+*δ*_, (*b*) Y_0.95_Ti_0.05_BaCo_4_O_7+*δ*_ and (*c*) Y_0.5_Dy_0.5_BaCo_4_O_7+*δ*_ samples.

The phase composition of the YBaCo_4_O_7+*δ*_, Y_0.95_Ti_0.05_BaCo_4_O_7+*δ*_ and Y_0.5_Dy_0.5_BaCo_4_O_7+*δ*_ samples is shown in figure 1. The cell parameters of the samples are refined from the data in space group *P*6_3_*mc*, and the refined cell parameters are presented in table 1. By combining with the XRD patterns and refined cell parameters, the present samples are indexed to be of YBaCo_4_O_7+*δ*_ structure. Typical SEM images of samples are shown in figure 2. It is seen that the samples consist of porous agglomerates of primary particles. The differences in morphology with different substituting ions of the oxygen carriers are very small.

**Table 1.** Refinement details for the samples.

sample	*a* (Å)	*b* (Å)	*c* (Å)	*V* (Å^3^)
YBaCo_4_O_7+*δ*_	6.2983	6.2983	10.1728	349.4661
Y_0.5_Dy_0.5_BaCo_4_O_7+*δ*_	6.3076	6.3076	10.1953	351.2900
Y_0.95_Ti_0.05_BaCo_4_O_7+*δ*_	6.2969	6.2969	10.1725	349.3005

**Figure 3.**
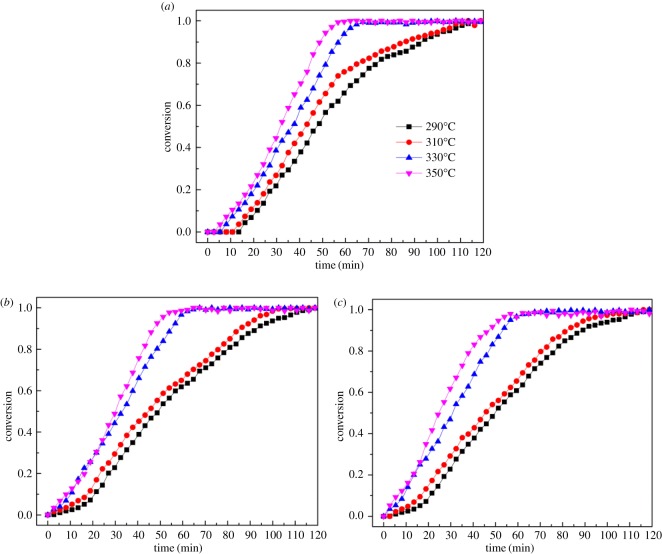
The conversion of oxidation in air atmosphere for the (*a*) YBaCo_4_O_7+*δ*_, (*b*) Y_0.95_Ti_0.05_BaCo_4_O_7+*δ*_ and (*c*) Y_0.5_Dy_0.5_BaCo_4_O_7+*δ*_ samples.

The 114 phase oxygen carriers can absorb certain amounts of oxygen at different temperatures. The percentage change in mass Δ*m* (%) and total stoichiometric change (*δ*) obtained at different oxidation temperatures are presented in table 2. The amount of oxygen absorption increases with increase in the oxidation temperature lower than 330°C. The amount of oxygen absorption obtained at 350°C is lower than that of the value obtained at 330 and 310°C. Furthermore, at a given oxidation temperature, the amount of oxygen absorption of Ti and Dy substituting samples is larger than that of the unsubstituted sample. Figure 3*a–c* shows the conversions of the YBaCo_4_O_7+*δ*_, Y_0.95_Ti_0.05_BaCo_4_O_7+*δ*_ and Y_0.5_Dy_0.5_BaCo_4_O_7+*δ*_ oxygen carriers during oxidation reactions at different temperatures, respectively. As can be seen, the oxygen absorption rate of oxygen carriers increases with increase in the oxidation temperature. The oxygen carriers absorb oxygen completely within 70 min when the temperatures are 330 and 350°C. When the temperatures are 290 and 310°C, the saturation time of oxygen adsorption is approximately 100 min. The reason behind this may be that increase in oxidation temperature is conducive to the greater diffusion of oxygen ions.

**Table 2.** The mass change of the samples under different temperatures.

	YBaCo_4_O_7+*δ*_				Y_0.95_Ti_0.05_BaCo_4_O_7+*δ*_				Y_0.5_Dy_0.5_BaCo_4_O_7+*δ*_			
	290°C	310°C	330°C	350°C	290°C	310°C	330°C	350°C	290°C	310°C	330°C	350°C
Δ*m*/%	1.982	2.211	2.244	2.123	2.011	2.341	2.376	2.209	1.897	2.289	2.315	2.237
*δ*	0.711	0.793	0.805	0.762	0.719	0.837	0.849	0.790	0.724	0.874	0.884	0.854

### 3.2. Kinetic models

The reaction rate of the process [45] can be written as follows:

3.1 $$k=\frac{\text{d}\alpha }{\text{d}t}=k(T)f(\alpha ),$$

where *α* is the extent of conversion, *k*(*T*) is the reaction rate content and *f*(*α*) is the kinetic model function. Equation (3.1) can be modified as follows:

3.2 $$\frac{\text{d}\alpha }{f(\alpha )}=k(T)\text{d}t.$$

Equation (3.2) can be transformed into equations (3.3*a*,*b*):

3.3a $$\int_{0}^{\alpha_{1}}\frac{\text{d}\alpha }{f(\alpha )}=G(\alpha )=\int_{0}^{t_{1}}k(T)\text{d}t$$

3.3b G(α)=k(T)t.

The plots of *G*(*α*) versus *t* should be straight lines whose slope can be used to determine the reaction rate *k*(*T*). The model showing the best linear fitting is chosen as the favoured model. The reaction models used for describing the oxidation process of oxygen carriers are presented in table 3 [41,42,45–49].

**Table 3.** Kinetic mechanism functions used for describing oxidation kinetics of oxygen carriers.

symbol	reaction model	*f*(*α*)	*g*(*α*)
R1	zero-order	1	*α*
R2	phase-boundary controlled reaction	2(1 − *α*)^1/2^	[1 − (1 − *α*)^1/2^]
R3	phase-boundary controlled reaction	3(1 − *α*)^2/3^	[1 − (1 − *α*)^1/3^]
F3/2	three-halves order	(1 − *α*)^3/2^	2[(1 − *α*)^−1/2^ − 1]
F2	second-order	(1 − *α*)^2^	(1 − *α*)^−1^ − 1
F3	third-order	(1 − *α*)^3^	(1/2)[(1 − *α*)^−2^ − 1]
A1/4	Avrami-Eroféev (*n* = 1/4)	(1/4)(1 − *α*)[−ln(1 − *α*)]^−3^	[−ln(1 − *α*)]^4^
A1/3	Avrami-Eroféev (*n* = 1/3)	(1/3)(1 − *α*)[−ln(1 − *α*)]^−2^	[−ln(1 − *α*)]^3^
A1/2	Avrami-Eroféev (*n* = 1/2)	(1/2)(1 − *α*)[−ln(1 − *α*)]^−1^	[−ln(1 − *α*)]^2^
A2/3	Avrami-Eroféev (*n* = 2/3)	(2/3)(1 − *α*)[−ln(1 − *α*)]^−1/2^	[−ln(1 − *α*)]^3/2^
A1	Avrami-Eroféev (*n* = 1)	(1 − *α*)	−ln(1 − *α*)
A3/2	Avrami-Eroféev (*n* = 3/2)	(3/2)(1 − *α*)[−ln(1 − *α*)]^1/3^	[−ln(1 − *α*)]^2/3^
A2	Avrami-Eroféev (*n* = 2)	2(1 − *α*)[−ln(1 − *α*)]^1/2^	[−ln(1 − *α*)]^1/2^
A3	Avrami-Eroféev (*n* = 3)	3(1 − *α*)[−ln(1 − *α*)]^2/3^	[−ln(1 − *α*)]^1/3^
A4	Avrami-Eroféev (*n* = 4)	4(1 − *α*)[−ln(1 − *α*)]^3/4^	[−ln(1 − *α*)]^1/4^

**Figure 4.**
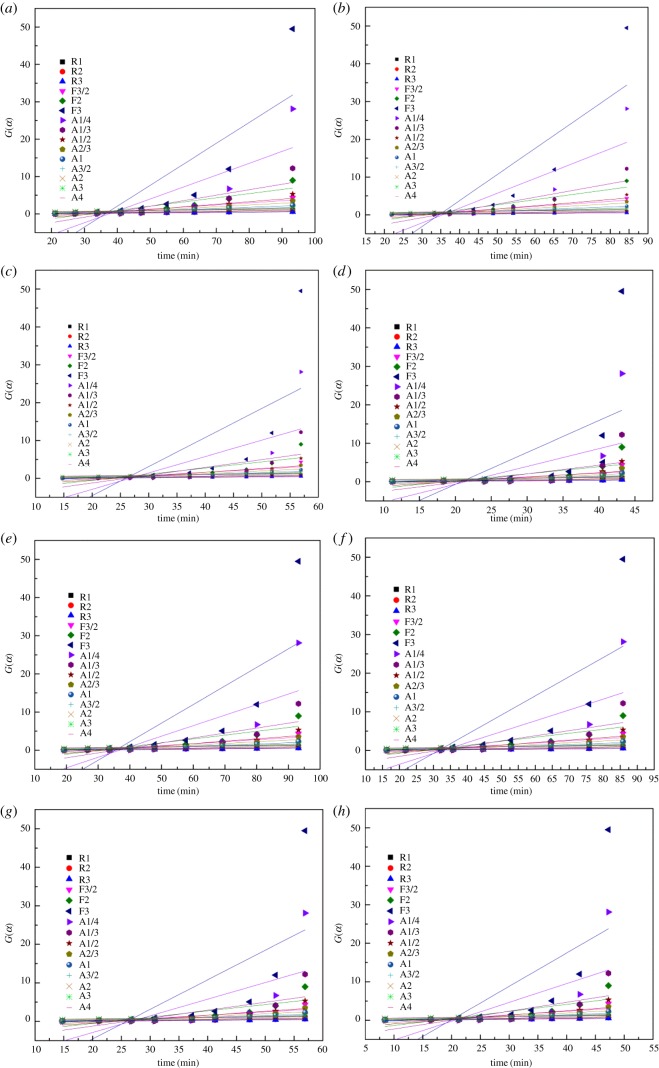

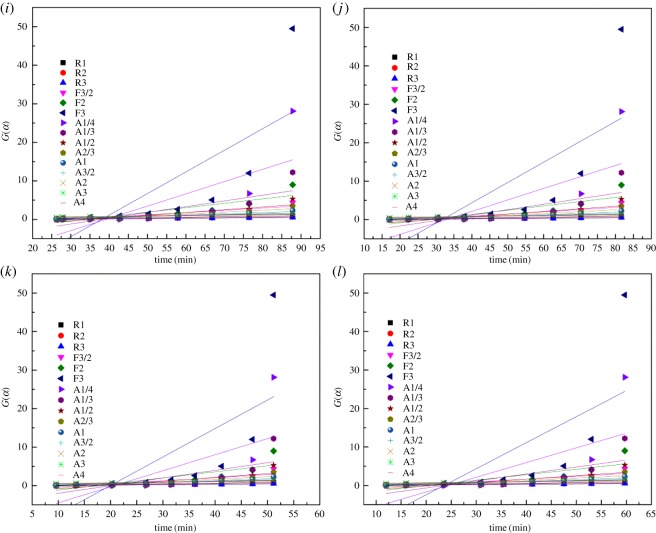
Trends of *G*(*α*) versus *t* under different temperatures with common mechanism functions for the YBaCo_4_O_7+*δ*_ oxidation process at (*a*) 290°C, (*b*) 310°C, (*c*) 330°C and (*d*) 350°C; for the Y_0.95_Ti_0.05_BaCo_4_O_7+*δ*_ oxidation process at (*e*) 290°C, (*f*) 310°C, (*g*) 330°C and (*h*) 350°C; and for the Y_0.5_Dy_0.5_BaCo_4_O_7+*δ*_ oxidation process at (*i*) 290°C, (*j*) 310°C, (*k*) 330°C and (*l*) 350°C.

By linear fitting the mechanism functions against *t* (parameters were estimated in the 0.1–0.90 conversion range), the linear correlation coefficient *R*^2^ and the residual sum of squares (RSS) of each function can be obtained. Figure 4 shows the fitting linear curves *G*(*α*) versus *t* under different oxidation temperatures.

Tables 4 and 5 list the *R*^2^ and RSS values obtained by fitting functions, respectively. The discrimination among the models was based on the higher *R*^2^ and lower RSS. The functions with the bigger *R*^2^ and smaller RSS values are selected as the mechanism functions. For the YBaCo_4_O_7+*δ*_ and Y_0.5_Dy_0.5_BaCo_4_O_7+*δ*_ samples, given the *R*^2^ and RSS values obtained, it was concluded that the A model and R better fitting were achieved with *n* values of 4 and 1, respectively. For the Y_0.95_Ti_0.05_BaCo_4_O_7+*δ*_ sample, it was concluded that the A model and R better fitting were achieved with *n* values of 3 and 1, respectively. For the Avrami-Eroféev random nucleation and the nuclei growth model, the overall conversion of the oxygen absorption reaction is determined by the relative rates of nucleation, nuclei growth and nucleus formation [24,50–52]. Nucleation and crystal growth are a dynamic process which practically initiates the oxygen absorption reaction. Generally, for the unreacted shrinking-core model, the overall conversion of the oxygen absorption reaction is determined by the chemical process [24]. That is, the overall conversion of the reaction is dominated by the chemical reaction, not the diffusion process for the A models and R models. The determined models can be used to evaluate the reaction rate, apparent activation energy and pre-exponential factor of the oxygen absorption reaction. For the YBaCo_4_O_7+*δ*_, Y_0.95_Ti_0.05_BaCo_4_O_7+*δ*_ and Y_0.5_Dy_0.5_BaCo_4_O_7+*δ*_ samples, the reaction rate constants are evaluated and presented in table 6.

**Table 4.** The linear correlation coefficient *R*^2^ of the mechanism models under different temperatures. The values in italics are the three largest values of *R*^2^ obtained by fitting functions.

	YBaCo_4_O_7+*δ*_					Y_0.95_Ti_0.05_BaCo_4_O_7+*δ*_					Y_0.5_Dy_0.5_BaCo_4_O_7+*δ*_				
code	290°C	310°C	330°C	350°C	average	290°C	310°C	330°C	350°C	average	290°C	310°C	330°C	350°C	average
R1	0.9657	0.9388	0.9984	0.9548	*0*.*9644*	0.9842	0.9862	0.9984	0.9920	*0*.*9902*	0.9833	0.9959	0.9975	0.9855	*0*.*9906*
R2	0.9962	0.9837	0.9729	0.8833	0.9590	0.9984	0.9943	0.9729	0.9599	0.9814	0.9961	0.9916	0.9773	0.9799	0.9862
R3	0.9978	0.9913	0.9542	0.8501	0.9484	0.9932	0.9868	0.9942	0.9400	0.9786	0.9903	0.9801	0.9605	0.9692	0.9750
F3/2	0.9260	0.9518	0.7883	0.6351	0.8253	0.8826	0.8648	0.7883	0.7740	0.8274	0.8769	0.8436	0.8040	0.8438	0.8421
F2	0.8358	0.8773	0.6330	0.5040	0.7125	0.7731	0.7505	0.6630	0.6521	0.7097	0.7667	0.7270	0.6824	0.7359	0.7280
F3	0.6635	0.7203	0.4680	0.3261	0.5445	0.5812	0.5546	0.4689	0.4648	0.5174	0.5749	0.5348	0.4905	0.5554	0.5389
A1/4	0.6446	0.7028	0.4482	0.3047	0.5251	0.5625	0.5362	0.4482	0.4439	0.4977	0.5558	0.5143	0.4699	0.5348	0.5187
A1/3	0.7314	0.7834	0.5392	0.3820	0.6090	0.6579	0.6331	0.5392	0.5304	0.5902	0.6507	0.6068	0.5601	0.6209	0.6092
A1/2	0.8560	0.8930	0.6890	0.5246	0.7406	0.8013	0.7803	0.6890	0.6741	0.7362	0.7948	0.7530	0.7071	0.7555	0.7526
A2/3	0.9270	0.9508	0.7910	0.6346	0.8258	0.8877	0.8705	0.7910	0.7740	0.8308	0.9896	0.8471	0.8061	0.8429	0.8714
A1	0.9851	0.9915	0.9006	0.7713	0.9121	0.9652	0.9540	0.9006	0.8852	0.9263	0.9611	0.9401	0.9110	0.9324	0.9362
A3/2	0.9994	0.9928	0.9619	0.8470	0.9503	0.9937	0.9877	0.9619	0.9517	0.9738	0.9896	0.9835	0.9676	0.9788	0.9799
A2	0.9939	0.9809	0.9827	0.7957	0.9383	0.9900	0.9919	0.9827	0.9767	0.9853	0.9802	0.9934	0.9856	0.9925	0.9879
A3	0.9778	0.9589	0.9937	0.9365	*0*.*9667*	0.9852	0.9850	0.9937	0.9931	*0*.*9893*	0.9796	0.9924	0.9935	0.9971	*0*.*9907*
A4	0.9654	0.9491	0.9949	0.9527	*0*.*9642*	0.9757	0.9769	0.9949	0.9974	*0*.*9862*	0.9694	0.9933	0.9939	0.9957	*0*.*9881*

**Table 5.** The residual sum of squares RSS of the mechanism models under different temperatures. The values in italics are the RSS values obtained by fitting three functions with the largest values of *R*^2^.

	YBaCo_4_O_7+*δ*_					Y_0.95_Ti_0.05_BaCo_4_O_7+*δ*_					Y_0.5_Dy_0.5_BaCo_4_O_7+*δ*_				
code	290°C	310°C	330°C	350°C	average	290°C	310°C	330°C	350°C	average	290°C	310°C	330°C	350°C	average
R1	0.0206	0.0367	0.0010	0.0271	*0*.*0214*	0.0095	0.0083	0.0097	0.0053	*0*.*0082*	0.0100	0.0025	0.0025	0.0027	*0*.*0044*
R2	0.0014	0.0058	0.0097	0.0419	0.0147	0.0006	0.0020	0.0097	0.0143	0.0067	0.0014	0.0030	0.0082	0.0072	0.0050
R3	0.0005	0.0019	0.0102	0.0333	0.0115	0.0015	0.0029	11.4687	0.0133	2.8716	0.0021	0.0044	0.0088	0.0068	0.0055
F3/2	1.0945	0.7120	3.1300	4.3932	2.3324	1.7352	1.9987	3.1300	3.3400	2.5509	1.8195	2.3126	2.8974	2.3086	2.3345
F2	10.683	7.983	21.921	32.264	18.2128	14.7584	16.230	21.921	22.628	18.884	15.180	17.762	20.661	17.181	17.696
F3	688.64	572.29	1086.69	1379.06	931.670	857.06	911.45	1086.69	1095.18	987.59	869.82	951.87	1042.48	909.84	943.50
A1/4	241.74	202.18	375.31	472.94	323.04	297.61	315.47	375.31	378.25	341.66	302.14	330.40	360.58	316.42	327.39
A1/3	34.464	27.789	59.124	79.289	50.166	43.888	47.079	59.124	60.248	52.585	44.810	50.447	56.438	48.640	50.08
A1/2	3.4898	2.5931	7.5353	11.5183	6.2842	4.1832	5.3235	7.5353	7.8953	6.2342	4.9724	5.9834	7.0962	5.9233	5.9938
A2/3	0.7566	0.5093	2.1652	3.7844	1.8039	1.1636	1.3416	2.1652	2.3407	1.7528	1.2197	1.5838	2.0081	1.6278	1.6099
A1	0.0617	0.0351	0.4113	0.9465	0.3637	0.1439	0.1902	0.4113	0.4748	0.3051	0.1610	0.2481	0.3685	0.2798	0.2644
A3/2	0.0011	0.0141	0.0745	0.2617	0.0879	0.0123	0.0240	0.0745	0.0943	0.0513	0.0203	0.0322	0.0633	0.0416	0.0394
A2	0.0073	0.0227	0.0206	0.1088	0.0399	0.0059	0.0097	0.0206	0.0276	0.0159	0.0115	0.0079	0.0172	0.0090	0.0114
A3	0.0313	0.0245	0.0076	0.0332	*0*.*0242*	0.0089	0.0090	0.0038	0.0041	*0*.*0065*	0.0122	0.0046	0.0039	0.0017	*0*.*0056*
A4	0.0125	0.0203	0.0019	0.0150	*0*.*0124*	0.0088	0.0084	0.0019	9.5628	*2*.*3955*	0.0111	0.0046	0.0026	0.0016	*0*.*0048*

**Table 6.** The reaction rate constants of the determined mechanism models under different temperatures.

	YBaCo_4_O_7+*δ*_				Y_0.95_Ti_0.05_BaCo_4_O_7+*δ*_					Y_0.5_Dy_0.5_BaCo_4_O_7+*δ*_				
code	290°C	310°C	330°C	350°C	code	290°C	310°C	330°C	350°C	code	290°C	310°C	330°C	350°C
A4	0.00813	0.01050	0.01488	0.01895	A3	0.00933	0.01130	0.01727	0.02165	A4	0.00744	0.00956	0.01452	0.01677
R1	0.01037	0.01348	0.01918	0.02434	R1	0.00929	0.01134	0.01918	0.02168	R1	0.00949	0.01236	0.01878	0.02148

From table 6, for the different mechanism functions and oxygen carriers, the reaction rate constant increases with increase in the reaction temperature, indicating that high temperature is propitious to the rate of oxygen adsorption. Low temperature may be one of the reasons accounting for the slow reaction rates shown in figure 3. Furthermore, the reaction rate (except for the reaction rate obtained at 290°C for the Y_0.95_Ti_0.05_BaCo_4_O_7+*δ*_ sample) obtained by the A model is lower than that of the R model. After evaluating the reaction rate constant, the pre-exponential factor and apparent activation energy can be evaluated.

Along with the Arrhenius expression, the following is obtained:

3.4 $$k(T)=A\exp\left(-\frac{E}{RT}\right),$$

where *A* is the pre-exponential factor, *E* is the apparent activation energy, *R* is the gas constant and *T* is the reaction temperature.

Along with the Arrhenius expression, the following form is obtained:

3.5 $$\ln k(T)=\ln A-\frac{E}{RT},$$

where the ln *k*(*T*) has been evaluated above, the plots ln *k*(*T*) versus 1/*T* are straight lines whose slope and intercept can be used to evaluate the apparent activation energy and pre-exponential factor, respectively.

**Figure 5.**
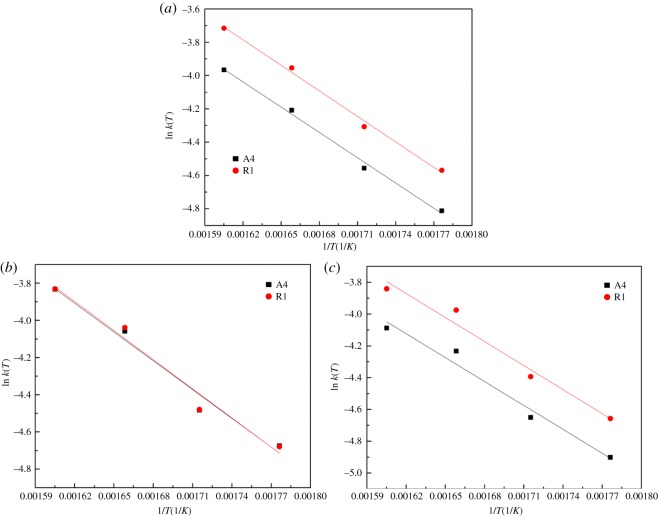
The plots of ln *k*(*T*) versus 1/*T* for different mechanism functions for the (*a*) YBaCo_4_O_7+*δ*_, (*b*) Y_0.95_Ti_0.05_BaCo_4_O_7+*δ*_ and (*c*) Y_0.5_Dy_0.5_BaCo_4_O_7+*δ*_ samples.

Figure 5 shows the plots ln *k*(*T*) versus 1/*T* as a function of different mechanism functions.

Table 7 lists the estimated apparent activation energy and pre-exponential factor as a function of reaction temperatures. The apparent activation energies obtained by the different mechanism functions remain close to constant levels for an oxygen carrier.

**Table 7.** The apparent activation energy and pre-exponential factor of the obtained function for the oxidation process of oxygen carriers.

oxygen carrier	code	*R*^2^	RSS	slope	intercept	*E* (J mol^−1^)	*A* (min^−1^)
YBaCo_4_O_7+*δ*_	A4	0.9948	0.00814	−4526.9800	3.2842	42078.5466	64.1677
	R1	0.9949	0.00853	−4522.3570	3.5280	42649.0491	92.2406
Y_0.95_Ti_0.05_BaCo_4_O_7+*δ*_	A3	0.9759	0.00977	−5165.2298	4.0446	42943.7206	84.8258
	R1	0.9739	0.01296	−5228.2237	4.5719	43467.4518	96.7277
Y_0.5_Dy_0.5_BaCo_4_O_7+*δ*_	A4	0.9739	0.01117	−5016.9196	4.0446	41710.6696	57.0901
	R1	0.9723	0.01234	−5040.2659	4.2952	41904.7707	73.3469

The activation energies for Y_0.5_Dy_0.5_BaCo_4_O_7+*δ*_ oxidation are found to be lower than those for YBaCo_4_O_7+*δ*_ and Y_0.95_Ti_0.05_BaCo_4_O_7+*δ*_ oxidation, thus confirming the favourable effect of Dy on the oxidizability of the YBaCo_4_O_7+*δ*_ oxygen carrier. This may be accounted for by the cell volume. The larger the cell volume, the easier is the absorption of oxygen. For an oxygen carrier, the pre-exponential factor obtained by the R model is larger than that of the A model.

For the purpose of further model discrimination between the A and R models, the A model is more favourable considering the higher unity of data values. In the case of the A model, the activation energies and the frequency factor remain close to constant levels at the different temperatures. Moreover, the activation energies with the R model vary in a much wider range. Thus, these results confirm the adequacy of the A model over the R model [24]. Thus, the nucleation and nuclei growth model is chosen as the most possible mechanism function.

The values of the established kinetic parameters, the apparent activation energies, the pre-exponential factors and the mechanism function were introduced into equation (3.1) and the differential equation was obtained to predict the oxygen carrier conversion of the oxidation process for different reaction time durations. The kinetic models are listed in table 8.

**Table 8.** The kinetic models of different oxygen carriers.

oxygen carrier	code	kinetic model
YBaCo_4_O_7+*δ*_	A4	dαdt=256.671exp⁡(−42078.547RT)∫(1−α)[−ln⁡(1−α)]3/4
Y_0.95_Ti_0.05_BaCo_4_O_7+*δ*_	A3	dαdt=254.477exp⁡(−42943.721RT)∫∫(1−α)[−ln⁡(1−α)]2/3
Y_0.5_Dy_0.5_BaCo_4_O_7+*δ*_	A4	dαdt=228.360exp⁡(−41710.670RT)∫∫(1−α)[−ln⁡(1−α)]3/4

## 4. Conclusion

In this work, kinetic behaviour of the oxidation process for the YBaCo_4_O_7+*δ*_, Y_0.95_Ti_0.05_BaCo_4_O_7+*δ*_ and Y_0.5_Dy_0.5_BaCo_4_O_7+*δ*_ oxygen carriers for CLAS operations was investigated for the temperature range of 290–350°C. The oxidation rate was found to increase gradually with increase in reaction temperature. It has been found that the A model provides a better description of the oxidation, indicating that the oxygen absorption process is rate-determined by nucleation and nuclei growth. The activation energies of the oxidation process obtained by the A model were determined as 42.079 kJ mol^−1^, 42.944 kJ mol^−1^ and 41.711 kJ mol^−1^ for the YBaCo_4_O_7+*δ*_, Y_0.95_Ti_0.05_BaCo_4_O_7+*δ*_ and Y_0.5_Dy_0.5_BaCo_4_O_7+*δ*_ oxygen carriers, respectively. The distributed activation energy of Y_0.5_Dy_0.5_BaCo_4_O_7+*δ*_ is lower than that of YBaCo_4_O_7+*δ*_, which corroborates the favourable effect of the substitution of Dy on the oxidizability of the oxygen carrier. The pre-exponential factors of the oxidation process obtained by the A model were determined as 64.168 min^−1^, 84.826 min^−1^ and 57.090 min^−1^ for the YBaCo_4_O_7+*δ*_, Y_0.95_Ti_0.05_BaCo_4_O_7+*δ*_ and Y_0.5_Dy_0.5_BaCo_4_O_7+*δ*_ oxygen carriers, respectively. The kinetic model was obtained to predict the oxygen carrier conversion of oxygen absorption for different time durations.

## Supplementary Material

Conversions of oxygen carriers during oxidation reaction under different temperatures

## Data accessibility

The data have been uploaded as the electronic supplementary file.
