# Supplementary material for: Oxidation kinetics of YBaCo4O7+δ and substituted oxygen carriers
Source: R Soc Open Sci. 2018 Jun 20;5(6):180150. doi: 10.1098/rsos.180150 (PMC6030326; doi:10.1098/rsos.180150)
Supplement: Conversions of oxygen carriers during oxidation reaction under different temperatures [file rsos180150supp1.docx]

Table 1 Conversions of YBaCo_4_O_7+δ_, Y_0.95_Ti_0.05_BaCo_4_O_7+δ_ and Y_0.5_Dy_0.5_BaCo_4_O_7+δ_ oxygen carriers during oxidation reaction under different temperatures

| Time/min | YBaCo_4_O_7+δ_ | | | | Y_0.95_Ti_0.05_BaCo_4_O_7+δ_ | | | | Y_0.5_Dy_0.5_BaCo_4_O_7+δ_ | | | |
| --- | --- | --- | --- | --- | --- | --- | --- | --- | --- | --- | --- | --- |
|  | 290/^○^C | 310/^○^C | 330/^○^C | 350/^○^C | 290/^○^C | 310/^○^C | 330/^○^C | 350/^○^C | 290/^○^C | 310/^○^C | 330/^○^C | 350/^○^C |
| 0 | 0 | 0 | 0 | 0 | 0 | 0 | 0 | 0 | 0 | 0 | 0 | 0 |
| 2.7 | 0 | 0 | 0 | 0 | 0.001 | 0.0165 | 0.0285 | 0.0342 | 0.001 | 0 | 0.0355 | 0.0528 |
| 5.4 | 0 | 0 | 0 | 0.0359 | 0.011 | 0.0231 | 0.0403 | 0.0685 | 0.0108 | 0.0229 | 0.0534 | 0.0929 |
| 8.1 | 0 | 0 | 0.0365 | 0.0721 | 0.019 | 0.0354 | 0.0691 | 0.0997 | 0.0192 | 0.0351 | 0.0841 | 0.1232 |
| 10.8 | 0 | 0 | 0.0732 | 0.1050 | 0.025 | 0.0518 | 0.1086 | 0.1281 | 0.0251 | 0.0481 | 0.1415 | 0.1619 |
| 13.5 | 0 | 0.0368 | 0.1065 | 0.1347 | 0.035 | 0.0693 | 0.1727 | 0.1611 | 0.0351 | 0.0687 | 0.2009 | 0.2064 |
| 16.2 | 0.044 | 0.0738 | 0.1367 | 0.1764 | 0.0516 | 0.0847 | 0.2262 | 0.1977 | 0.0516 | 0.0949 | 0.2505 | 0.2625 |
| 18.9 | 0.0682 | 0.1074 | 0.1790 | 0.2165 | 0.0718 | 0.1162 | 0.2561 | 0.2554 | 0.0718 | 0.1326 | 0.2790 | 0.3500 |
| 21.6 | 0.1022 | 0.1379 | 0.2198 | 0.2689 | 0.1122 | 0.1694 | 0.3003 | 0.3047 | 0.1122 | 0.1735 | 0.3268 | 0.4168 |
| 24.3 | 0.136 | 0.1805 | 0.2730 | 0.3206 | 0.1436 | 0.2220 | 0.3452 | 0.3612 | 0.1456 | 0.2170 | 0.3625 | 0.4920 |
| 27 | 0.1931 | 0.2366 | 0.3149 | 0.3904 | 0.2019 | 0.2514 | 0.3915 | 0.4338 | 0.1931 | 0.2494 | 0.4328 | 0.5528 |
| 29.7 | 0.2181 | 0.2678 | 0.3858 | 0.4443 | 0.2281 | 0.2947 | 0.4430 | 0.4885 | 0.2281 | 0.2921 | 0.4694 | 0.6172 |
| 32.4 | 0.269 | 0.3140 | 0.4322 | 0.5212 | 0.2769 | 0.3388 | 0.4878 | 0.5713 | 0.2769 | 0.3273 | 0.5426 | 0.6711 |
| 35.1 | 0.2941 | 0.3761 | 0.4711 | 0.5875 | 0.3141 | 0.3842 | 0.5346 | 0.6212 | 0.3141 | 0.3810 | 0.5829 | 0.7297 |
| 37.8 | 0.3349 | 0.4197 | 0.5113 | 0.6524 | 0.3491 | 0.4228 | 0.5989 | 0.6871 | 0.3491 | 0.3987 | 0.6273 | 0.7900 |
| 40.5 | 0.3791 | 0.4633 | 0.5887 | 0.7039 | 0.3891 | 0.4523 | 0.6606 | 0.7570 | 0.3791 | 0.4291 | 0.6876 | 0.8314 |
| 43.2 | 0.4211 | 0.5100 | 0.6256 | 0.7588 | 0.4311 | 0.4817 | 0.7146 | 0.8176 | 0.4211 | 0.4681 | 0.7485 | 0.8678 |
| 45.9 | 0.478 | 0.5634 | 0.6796 | 0.8413 | 0.4678 | 0.5146 | 0.7631 | 0.8820 | 0.4478 | 0.5060 | 0.7789 | 0.8908 |
| 48.6 | 0.5130 | 0.6153 | 0.7410 | 0.8975 | 0.4930 | 0.5526 | 0.8023 | 0.9255 | 0.4830 | 0.5407 | 0.8319 | 0.9170 |
| 51.3 | 0.566 | 0.6556 | 0.7923 | 0.9431 | 0.5366 | 0.5873 | 0.8393 | 0.9560 | 0.5237 | 0.5605 | 0.8682 | 0.9469 |
| 54 | 0.5984 | 0.6970 | 0.8529 | 0.9706 | 0.5635 | 0.6133 | 0.8844 | 0.9772 | 0.5535 | 0.5926 | 0.9116 | 0.9661 |
| 56.7 | 0.6188 | 0.7392 | 0.8967 | 0.9927 | 0.5988 | 0.6322 | 0.9244 | 0.9795 | 0.5878 | 0.6241 | 0.9501 | 0.9806 |
| 59.4 | 0.6582 | 0.7587 | 0.9376 | 0.9962 | 0.6182 | 0.6499 | 0.9667 | 0.9854 | 0.6082 | 0.6546 | 0.9669 | 0.9638 |
| 62.1 | 0.6936 | 0.7734 | 0.9652 | 0.9992 | 0.6361 | 0.6795 | 0.9832 | 0.9901 | 0.6361 | 0.6939 | 0.9778 | 0.9839 |
| 64.8 | 0.7155 | 0.7951 | 0.9829 | 1.0000 | 0.6551 | 0.7023 | 0.9916 | 0.9999 | 0.6785 | 0.7328 | 0.9798 | 0.9820 |
| 67.5 | 0.7438 | 0.8099 | 0.9940 | 1.0000 | 0.6944 | 0.7244 | 1.0000 | 0.9999 | 0.7144 | 0.7562 | 0.9888 | 0.9852 |
| 70.2 | 0.7750 | 0.8230 | 0.9905 | 1.0000 | 0.7103 | 0.7447 | 1.0000 | 0.9901 | 0.7410 | 0.7978 | 0.9877 | 0.9884 |
| 72.9 | 0.7929 | 0.8388 | 0.9899 | 1.0000 | 0.7288 | 0.7759 | 1.0000 | 0.9934 | 0.7688 | 0.8190 | 0.9927 | 0.9727 |
| 75.6 | 0.8181 | 0.8562 | 0.9930 | 1.0000 | 0.7581 | 0.7983 | 1.0000 | 0.9836 | 0.7908 | 0.8575 | 0.9870 | 1.0000 |
| 78.3 | 0.8315 | 0.8657 | 0.9922 | 1.0000 | 0.7832 | 0.8259 | 1.0000 | 0.9999 | 0.8232 | 0.8686 | 0.9953 | 0.9822 |
| 81 | 0.8391 | 0.8775 | 0.9919 | 1.0000 | 0.8091 | 0.8504 | 1.0000 | 0.9855 | 0.8491 | 0.8929 | 0.9898 | 0.9820 |
| 83.7 | 0.8490 | 0.8928 | 0.9922 | 1.0000 | 0.8249 | 0.8841 | 1.0000 | 0.9999 | 0.8649 | 0.9135 | 0.9970 | 0.9722 |
| 86.4 | 0.8561 | 0.9026 | 0.9829 | 1.0000 | 0.8561 | 0.9059 | 1.0000 | 0.9966 | 0.8856 | 0.9263 | 0.9912 | 0.9817 |
| 89.1 | 0.8754 | 0.9143 | 0.9905 | 1.0000 | 0.8754 | 0.9223 | 1.0000 | 0.9999 | 0.9018 | 0.9445 | 0.9973 | 0.9880 |
| 91.8 | 0.8898 | 0.9225 | 0.9940 | 1.0000 | 0.8898 | 0.9406 | 1.0000 | 0.9999 | 0.9198 | 0.9570 | 0.9907 | 0.9789 |
| 94.5 | 0.9125 | 0.9297 | 0.9940 | 1.0000 | 0.9125 | 0.9617 | 1.0000 | 0.9938 | 0.9252 | 0.9633 | 0.9972 | 0.9911 |
| 97.2 | 0.9251 | 0.9402 | 0.9975 | 1.0000 | 0.9251 | 0.9674 | 1.0000 | 0.9999 | 0.9351 | 0.9704 | 0.9903 | 0.9684 |
| 99.9 | 0.9363 | 0.9455 | 0.9975 | 1.0000 | 0.9363 | 0.9841 | 1.0000 | 0.9868 | 0.9396 | 0.9731 | 0.9870 | 0.9880 |
| 102.6 | 0.9496 | 0.9576 | 0.9940 | 1.0000 | 0.9496 | 0.9949 | 1.0000 | 0.9999 | 0.9496 | 0.9804 | 0.9935 | 0.9911 |
| 105.3 | 0.9525 | 0.9650 | 0.9940 | 1.0000 | 0.9525 | 0.9955 | 1.0000 | 0.9839 | 0.9525 | 0.9802 | 0.9967 | 0.9939 |
| 108 | 0.9655 | 0.9858 | 1.0000 | 1.0000 | 0.9655 | 0.9967 | 1.0000 | 0.9998 | 0.9655 | 0.9807 | 0.9903 | 0.9848 |
| 110.7 | 0.978 | 0.9895 | 1.0000 | 1.0000 | 0.978 | 0.9977 | 1.0000 | 0.9868 | 0.9781 | 0.9877 | 0.9870 | 1.0000 |
| 113.4 | 0.9863 | 1.0000 | 1.0000 | 1.0000 | 0.9863 | 0.9987 | 1.0000 | 0.9999 | 0.9863 | 0.9866 | 0.9935 | 1.0000 |
| 116.1 | 0.9901 | 1.0000 | 1.0000 | 1.0000 | 0.9901 | 0.9993 | 1.0000 | 0.9868 | 0.9901 | 0.9992 | 0.9902 | 1.0000 |
| 118.8 | 1.0000 | 1.0000 | 1.0000 | 1.0000 | 1.0000 | 1.0000 | 1.0000 | 1.0000 | 1.0000 | 1.0000 | 1.0000 | 1.0000 |
